# Activation of Peroxisome Proliferator-Activated Receptor Alpha Improves Aged and UV-Irradiated Skin by Catalase Induction

**DOI:** 10.1371/journal.pone.0162628

**Published:** 2016-09-09

**Authors:** Mi Hee Shin, Se-Rah Lee, Min-Kyoung Kim, Chang-Yup Shin, Dong Hun Lee, Jin Ho Chung

**Affiliations:** 1 Department of Dermatology, Seoul National University College of Medicine, Seoul, Republic of Korea; 2 Biomedical Research Institute, Seoul National University Hospital, Seoul, Republic of Korea; 3 Institute of Human-Environment Interface Biology, Medical Research Center, Seoul National University, Seoul, Republic of Korea; 4 Institute on Aging, Seoul National University, Seoul, Republic of Korea; University of Alabama at Birmingham, UNITED STATES

## Abstract

Peroxisome proliferator-activated receptor alpha (PPARα) is a nuclear hormone receptor involved in the transcriptional regulation of lipid metabolism, fatty acid oxidation, and glucose homeostasis. Its activation stimulates antioxidant enzymes such as catalase, whose expression is decreased in aged human skin. Here we investigated the expression of PPARα in aged and ultraviolet (UV)-irradiated skin, and whether PPARα activation can modulate expressions of matrix metalloproteinase (MMP)-1 and procollagen through catalase regulation. We found that PPARα mRNA level was significantly decreased in intrinsically aged and photoaged human skin as well as in UV-irradiated skin. A PPARα activator, Wy14643, inhibited UV-induced increase of MMP-1 and decrease of procollagen expression and caused marked increase in catalase expression. Furthermore, production of reactive oxygen species (ROS) was suppressed by Wy14643 in UV-irradiated and aged dermal fibroblasts, suggesting that the PPARα activation-induced upregulation of catalase leads to scavenging of ROS produced due to UV irradiation or aging. PPARα knockdown decreased catalase expression and abolished the beneficial effects of Wy14643. Topical application of Wy14643 on hairless mice restored catalase activity and prevented MMP-13 and inflammatory responses in skin. Our findings indicate that PPARα activation triggers catalase expression and ROS scavenging, thereby protecting skin from UV-induced damage and intrinsic aging.

## Introduction

Skin aging is thought to occur through two processes: intrinsic aging and photoaging. Photoaging is caused by chronic ultraviolet (UV)-induced damage during the aging process. Photoaging and intrinsic aging share important molecular features, including altered signal transduction pathways that promote matrix metalloproteinase (MMP) expression and decrease procollagen synthesis. Alterations in collagen, the major structural component of the skin, have been suggested to cause clinical changes of the skin such as wrinkling and loss of elasticity, which are generally observed in intrinsically aged and photoaged skin. UV irradiation induces the synthesis of various MMPs, such as MMP-1, -3, -9, and -12, in human skin, and MMP-mediated collagen destruction accounts for the majority of connective tissue damage during photoaging [1–3]. In addition, recent studies indicate that UV exposure modulates neuroendocrine homeostasis [4, 5] and steroidogenesis in the skin [6], in a wavelength-dependent manner [7].

Acute exposure of UV radiation leads to inflammatory responses in the skin [8]. UV induces the expression of a diverse array of proinflammatory mediators, such as interleukin (IL)-1β, IL-6, IL-8 and tumor necrosis factor (TNF)-α, via the activation of transcription factor NF-κB in human skin *in vivo* [9, 10]. Cyclooxygenase (COX)-2 has also been shown to have central roles in UV-stimulated acute inflammation [11, 12]. Inflammation exacerbates the aging process by various mechanisms such as the overproduction of free radicals [13, 14].

Furthermore, UV radiation causes ROS production and depletion of antioxidant enzymes in the human skin. Catalase has been recognized as the most important antioxidant enzyme implicated in human skin aging [15]. Previously, we demonstrated that acute UV radiation gradually decreases the activity and expression of catalase in the human skin *in vivo* [16]. Decreased catalase expression may cause ROS accumulation, and ROS has been shown to upregulate MMPs and downregulate collagen synthesis, thus resulting in accelerated intrinsic aging and photoaging of the human skin [17, 18].

Peroxisome proliferator-activated receptors (PPARs) are ligand-activated transcription factors that belong to a family of nuclear hormone receptors and consist of three isotypes (PPARα, β/δ, and γ). These PPAR isotypes share high degree of structural homology but differ in their functional roles and tissue expression. Although PPARβ/δ is the predominant isotype found in the skin, PPARα is also expressed in the skin, as well as in various tissues such as the liver, brown adipose tissue, heart, and kidney [19]. PPARα has an important role in the fatty acid oxidation, lipid and lipoprotein metabolism, inflammatory responses, and oxidative stress [20, 21]. PPARα expression is important for the maintenance of cellular redox balance. An antioxidant enzyme, catalase, is a known target of PPARα and the activation of PPARα using agonists has been reported to enhance the superoxide dismutase expression and catalase activity in mouse liver [22–24]. Moreover, PPARα activators were recently shown to inhibit the expression of MMPs, IL-6, and IL-8 via activating protein-1 (AP-1) and NF-κB pathways for their anti-inflammatory and anti-aging effects [25].

In this study, we investigated PPARα mRNA expression in intrinsically aged and photoaged skins and investigated whether activation of PPARα by an agonist can prevent UV-induced skin damages in human dermal fibroblasts and hairless mice. Our results demonstrate that ligand-activated PPARα was able to attenuate UV-induced secretion of MMP-1 and prevent UV-induced suppression of procollagen expression through ROS inhibition mediated by catalase induction.

## Materials and Methods

### Cell culture, UV irradiation and Wy14643 treatment

Primary human dermal fibroblasts were cultured in Dulbecco's modified eagle medium (DMEM) supplemented with 2 mM glutamine, penicillin (100 U/ml), streptomycin (100 μg/ml) and 10% heat-inactivated fetal bovine serum (FBS) at 37°C and 5% CO_2_.

Philips F75/85W/UV21 fluorescent sun lamps (Einthoven, Netherlands) with an emission spectrum between 275 and 380 nm were used as the UV source [26]. The distribution of their power output was 56.7% UVB (280–320 nm), 42.8% UVA (320–400nm), and 0.5% UVC (<280 nm). A Kodacel filter (TA401/407; Kodak, Rochester, NY) was used to eliminate UVC. The UV intensity was measured using a UV photometer (Model 585100; Waldmann Co., Villingen-Schwenningen, Germany). Wy14643 was purchased from Sigma-Aldrich (St Louis, MO) and was first dissolved in dimethylsulfoxide (DMSO) and subsequently diluted with DMEM (final DMSO concentration, 0.1%).

### Human skin samples

Skin samples from sun-protected upper-inner arm skin and photodamaged forearm skin were obtained from young (3 men, mean age 21.6 year, age range 19–29 year) and elderly Korean volunteers (3 men, mean age 76.8 year, age range 70–83 year) without current or prior skin diseases. In addition, to investigate effects of UV in human skin *in vivo*, buttock skin of volunteers (9 men, mean age, 43.8 year; age range, 35–52 year), were irradiated with 2 MED (minimal erythema dose) of UV. At 24 h post-UV, non-irradiated and UV-irradiated skin was obtained. This study was conducted according to the Principles described in the Declaration of Helsinki. All experimental procedures received prior approval from the Institutional Review Board at Seoul National University Hospital. And written informed consent from the volunteers were obtained.

### Quantitative real-time RT-PCR

Total RNA was prepared from fibroblast or mouse skin sample using RNAiso Plus (Takara Bio Inc., Otsu, Shiga, Japan) and 1 μg of total RNA was converted to cDNA using a First Strand cDNA Synthesis Kit (MBI Fermentas, Vilnius, Lithuania). Quantitative PCR was performed using a 7500 Real-time PCR System (Applied Biosystems, Foster City, CA) and SYBR Green PCR Master Mix (Takara Bio, Inc.). Data were calculated using the 2^-ΔΔC[T]^ methods. Fold changes were calculated as the ratio of gene expression in each group relative to that in the control group.

### Western blot analysis

Western blotting was performed as described previously [27]. Briefly, to determine the amount of procollagen and MMP-1 secreted into the culture media, equal aliquots of conditioned culture media from an equal number of cells were fractionated by 10% SDS PAGE. Soluble protein fractions were extracted either from cultured fibroblasts or from mouse skin samples using RIPA buffer (Millipore, Billerica, MA). Lysates were centrifuged at 12,000xg for 10 min, and the supernatant was used for western blot analysis. The protein concentrations were determined by Bradford assay. Equal amounts of protein samples were separated and transferred. Blotting proteins were detected using ECL (enhanced chemiluminescence) system (Amersham, Buckinghamshire, UK). A monoclonal anti-MMP-1 antibody (Lab Frontier, Seoul, Korea), a monoclonal anti-α1(I) procollagen amino terminal extension peptide (SP1.D8) antibody (Developmental Studies Hybridoma Bank, Iowa City, IA), catalase (Calbiochem-Novabiochem, La Jolla, CA) and β-actin (Santa Cruz Biotechnology, Santa Cruz, CA) were used as primary antibodies. The band intensity was quantified using Bio 1D software (VilberLourmat, Marne La Vallec, France), normalized by that of β-actin, and compared relative to those of the control group as 100%.

### Catalase activity assay

Each sample of fibroblasts or mouse skin was homogenized in buffer A (pH 7.0, NaCl 130 mM, Na_2_HPO_4_ 10 mM, glucose 5 mM, and EDTA 1 mM). The homogenate was rotated at 4°C for 10 min, and centrifuged at 14,000 g for 10 min. Catalase activity was assayed using the supernatant on a Beckman DU650 Spectrophotometer (Beckman Instruments, Fullerton, CA), as previously described [15].

### Gene silencing with siRNA

Knockdown of human PPARα expression was performed by RNA interference method. The siRNA specific for PPARα (No.1120110) and control scrambled siRNA was obtained from Bioneer (Daejeon, Korea). Fibroblasts were transfected with negative control siRNA or PPARα siRNA at the indicated concentrations using Lipofectamine 2000 (Invitrogen, Carlsbad, CA).

### Measurement of intracellular H_2_O_2_

Intracellular H_2_O_2_ levels were measured by quantifying fluorescence of 2,7-dichlororofluorescein diacetate (DCFDA, Molecular Probes, Eugene, OR). Cells were cultured in DMEM until confluency of 80%. After serum-starvation for 24 h, DCFDA (25 μM) was treated for 30 min. Intracellular H_2_O_2_ levels were quantified using a fluorescence reader (an excitation wavelength of 488 nm and an emission wavelength of 515 nm).

### Animal experiment

Animal experimental procedure was performed as described previously [28, 29]. Six-week-old female albino hairless mice (Skh-1) were purchased from BioGenomics, Inc. (Seoul, Korea), acclimated for 1 week prior to the study commencement, and subject to UV irradiation. All experimental procedures were approved by the Institutional Animal Care and Use Committee (IACUC) of Seoul National University Hospital (Association for Assessment and Accreditation of Laboratory Animal Care (AAALAC)-accredited facility). F75/85W/UV21 fluorescent sunlamps were employed for UV irradiation with a Kodacel filter. The minimal erythema dose (MED) was firstly determined, and UV was irradiated to the back skin of hairless mice in 2 MED (1 MED = 100 mJ/cm^2^). Animals were divided into six groups as follows: (i) vehicle-treated group, (ii) 2 mM Wy14643-treated group, (iii) 10 mM Wy14643-treated group, (iv) UV-irradiated and vehicle-treated group, (v) UV-irradiated and 2 mM Wy14643-treated group, (vi) UV-irradiated and 10 mM Wy14643-treated group. Vehicle was composed of polyethylene glycol (70%) and ethanol (30%). Wy14643 or vehicle was topically applied to the dorsal skin surface at 24 hr before UV irradiation, and at 0 and 24 h after UV irradiation. When treated, the mice were anesthetized by an intraperitoneal injection of ketamine and xylazine. At 48 h after UV irradiation, these mice were sacrificed using lethal doses of ketamine and xylazine, followed by cervical dislocation, and skin samples were obtained.

### Statistical analysis

Statistical analyses were carried out using Student’s t-test. *P*-values of less than 0.05 were regarded as statistically significant.

## Results

### PPARα mRNA level was decreased in aged and photoaged human skin and in UV-irradiated skin *in vivo*

**Fig 1:**
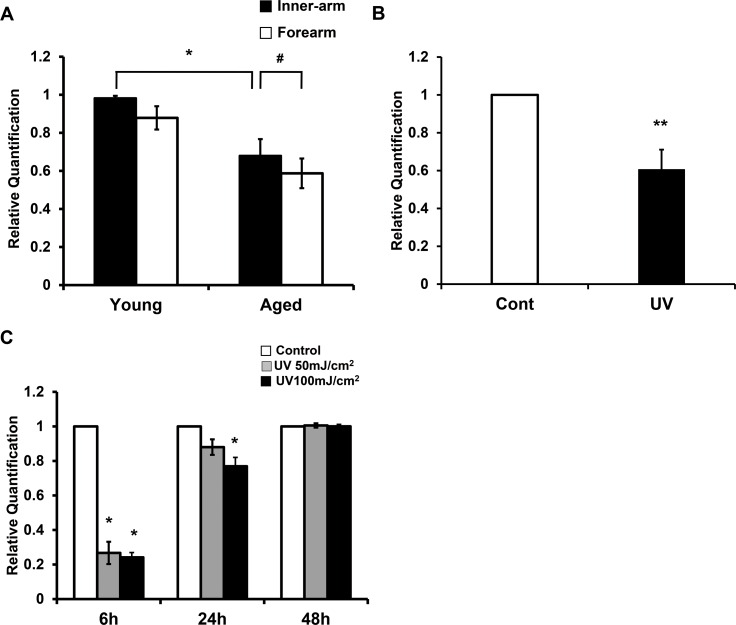
PPARα expression was decreased in aged and photoaged human skin and in UV-irradiated skin *in vivo* and in dermal fibroblasts. **(a)** Levels of PPARα mRNA in inner arms and forearms of young and elderly subjects were determined by qPCR. Data are presented as means±SEM (n = 3), *p<0.05 vs. inner arms of young subjects; #*p*<0.05 vs. inner-arms of old subjects. **(b)** Young buttock skin was irradiated with UV light, and obtained after 24 h. PPARα mRNA expression following UV irradiation was compared with that of non-irradiated skin (control) by qPCR. Data are presented as means±SEM (n = 9), **p<0.01 vs. non-irradiated skin. **(c)** Cultured fibroblasts were starved with serum-free DMEM for 24 h, irradiated with UV (50 or 100 mJ/cm^2^), and harvested at the indicated time points. Levels of PPARα mRNA following UV irradiation were compared to those of non-irradiated controls. Data are presented as means±SEM (n = 3), *p<0.05 vs. time-matched control.

To investigate effects of photoaging and intrinsic aging on PPARα mRNA expression in human skin *in vivo*, levels of PPARα mRNA in forearms and upper-inner arms of young and elderly subjects were assessed and compared using quantitative reverse transcription PCR (qPCR). We found that PPARα level in the inner-arm skin of elderly subjects was significantly reduced compared to that of young subjects (68.0±8.7%; *p*<0.05; n = 3) (**Fig 1A**). Additionally, PPARα mRNA expression in forearm skin of the elderly was significantly reduced compared to its expression in the inner-arm skin of the same individuals (*p*<0.05; n = 3). However, in young individuals, PPARα expression in the inner-arm skin was comparable to its expression in the forearm skin (**Fig 1A**). Therefore, the difference in PPARα expression is attributed to a photoaging event, but not difference in anatomical site. To observe the effect of acute UV irradiation on PPARα mRNA level, young buttock skin was irradiated with 2MED of UV light. As shown in **Fig 1B**, acute UV irradiation significantly decreased PPARα mRNA expression to 60.5±10.5% relative to non-irradiated skin of the same individuals (*p*<0.01; n = 9). In human dermal fibroblasts, we also observed a significant decrease in PPARα mRNA at 6 h post-UV irradiation, which gradually recovered over time (**Fig 1C**). Taken together, these results indicated that intrinsic aging, photoaging, as well as acute UV irradiation decreased PPARα expression in the human skin.

### PPARα agonist, Wy14643, was able to limit procollagen reduction and MMP-1 expression induced by UV in human dermal fibroblasts

**Fig 2:**
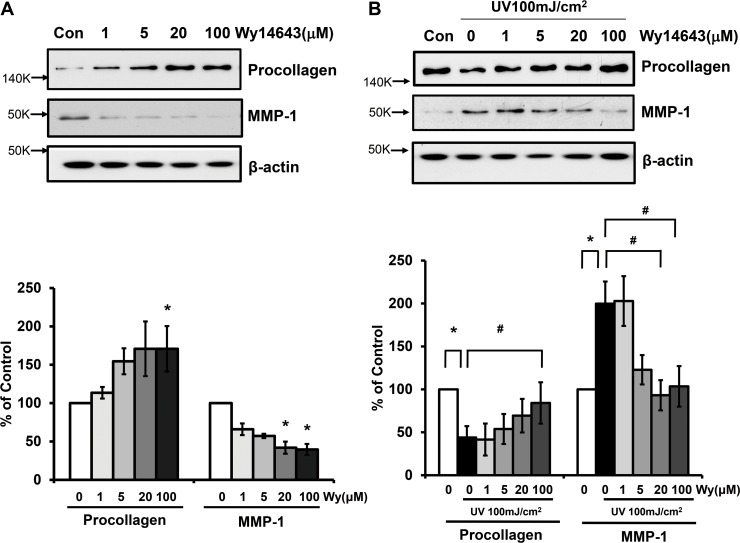
PPARα activator, Wy14643, could limit type I procollagen reduction and MMP-1 expression induced by UV in human dermal fibroblasts. **(a)** Human dermal fibroblasts were treated with 1–100 μM Wy14643 for 72 h. Western blot analysis using the culture media was performed to determine the expression of procollagen and MMP-1 while cell lysates were used to determine β-actin protein level as a loading control. **(b)** Cultured fibroblasts were exposed to UV irradiation (100 mJ/cm^2^). Following irradiation, cells were treated with 1–100μM Wy14643 for 72 h. The western bands are representative of three separate experiments. Data are presented as means±SEM (n = 3), *p<0.05 vs. control; #*p*<0.05 vs. UV-irradiated cells.

We investigated whether ligand-activated PPARα affects the basal expression of type I procollagen and MMP-1 in human dermal fibroblasts. Using western blot analyses, we demonstrated that Wy14643 significantly increased the expression of type I procollagen and decreased MMP-1 protein level in a dose-dependent manner (*p*<0.05; n = 3) (**Fig 2A**). To determine whether Wy14643 can exert any inhibitory effect on procollagen and MMP-1 modulations induced by UV, cells were irradiated with 100 mJ/cm^2^ of UV in the presence or absence of Wy14643 treatment. Cells treated with Wy14643 (100 μM) immediately following UV irradiation showed significant recovery of type I procollagen expression compared to vehicle-treated UV-irradiated cells. In contrast, MMP-1 expression induced by UV was significantly abrogated by Wy14643 in a dose-dependent manner (**Fig 2B**).

### PPARα agonist increased the expression of catalase and ameliorated UV-induced ROS production in human dermal fibroblasts

**Fig 3:**
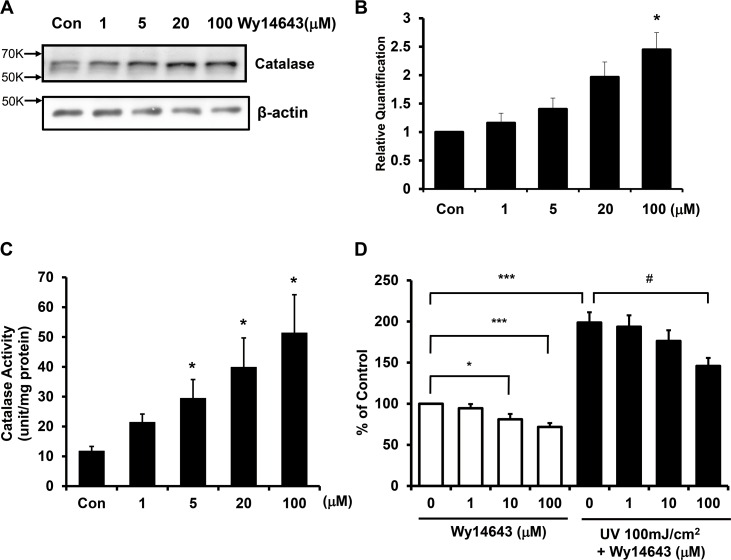
PPARα agonist increased the expression of catalase and ameliorated UV-induced ROS generation in human dermal fibroblasts. Cultured fibroblasts were serum-starved for 24 h, and treated with 1–100 μM Wy14643 for 72 h. **(a)** Total soluble proteins were isolated from the cells, and levels of catalase protein were measured by western blot analysis and normalized to β-actin. **(b)** Catalase mRNA expression was determined by qPCR. Data are presented as means±SEM (n = 3), *p<0.05 vs. control. **(c)** Catalase activity was measured by a spectrophotometric method. Catalase enzyme unit is equivalent to 1 μmol of substrate disappearance or product formation per min. Data are presented as mean±SEM (n = 3). **(d)** Human dermal fibroblasts were treated with Wy14643 for 72 h, and then irradiated with UV. Subsequently, cells were treated with 2,7-dichlororofluorescein diacetate (DCFDA) and assayed using a fluorescence reader. Data are presented as means±SEM (n = 6), *p<0.05, ***p<0.001 vs. control, #*p*<0.05 vs. UV-treated cells.

Since activation of PPARα by agonists is known to enhance catalase expression [22], we investigated the effect of Wy14643 on catalase expression in human dermal fibroblasts. Wy14643 significantly increased catalase protein and mRNA levels in a dose-dependent manner compared to the vehicle-treated control (**Fig 3A and 3B**). Wy14643 treatment was also found to significantly increase the catalase activity in a dose-dependent manner (**Fig 3C**). Taken together, ligand-activated PPARα increased the expression and activity of catalase in human dermal fibroblasts.

UV was reported to induce ROS generation, and ROS can lead to skin aging via various signaling pathways. To assess whether catalase upregulation by a PPARα agonist can lead to ROS scavenging, we examined the effect of Wy14643 on intracellular and UV-induced ROS levels. We found that Wy14643 reduced basal level of ROS in a dose-dependent manner. Upon UV irradiation, ROS generation was markedly increased compared to the control (198.7±12.6%). However, cells treated with 100 μM Wy14643 showed significant inhibition of ROS production induced by UV when compared to untreated UV-irradiated fibroblasts (**Fig 3D**). These results indicated that catalase expression was increased by ligand-activated PPARα, and the elevated catalase was able to limit the generation of ROS induced by UV.

### PPARα small interfering RNAs (siRNA) transfection decreased catalase expression and abolished beneficial effects of Wy14643 in human dermal fibroblasts

**Fig 4:**
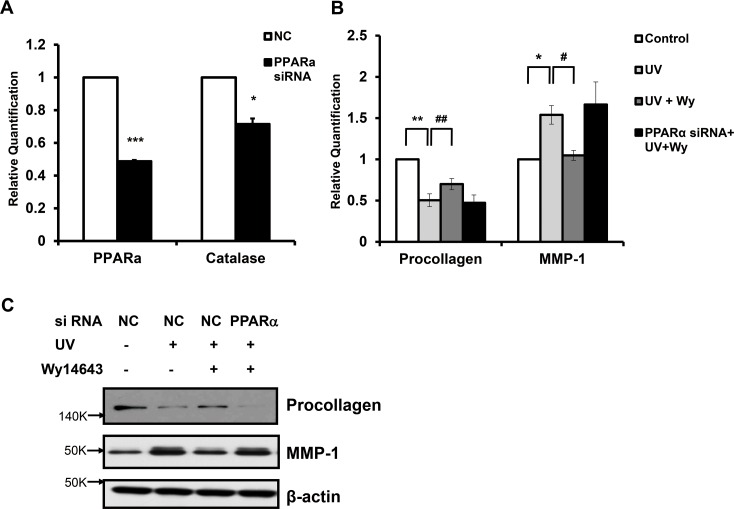
PPARα siRNA transfection decreased catalase expression and abolished beneficial effects of Wy14643 in human dermal fibroblasts. **(a)** Human fibroblasts were transfected with scrambled siRNA as a negative control (NC) or siRNAs selectively targeting PPARα (100 nM) for 48 h. Cells were subjected to serum-starvation for 24 h. Levels of PPARα and catalase mRNAs were measured by qPCR. Data are presented as mean±SEM (n = 3), *p<0.05, ***p<0.001 vs. cells transfected with scrambled siRNA **(b)** Cultured fibroblasts were transfected with scrambled siRNA or PPARα siRNA for 48 h, and serum-starved for 24 h. Then, they were exposed to UV irradiation (100 mJ/cm^2^), followed by 1–100 μM Wy14643 treatment for 72 h. Levels of procollagen and MMP-1 proteins released into culture media were measured by western blotting. The bands are representative of results from three independent experiments. **(c)** Procollagen and MMP-1 mRNA expression was measured by qPCR. Data are presented as mean±SEM (n = 3), *p<0.05, **p<0.01 vs. UV-treated cells; #*p*<0.05, ##*p*<0.01 vs. NC siRNA-transfected UV-treated cells.

In order to confirm the role of PPARα in modulating the expression of procollagen and MMP-1 induced by UV in human dermal fibroblasts, we utilized siRNA to knockdown the expression of PPARα. We transfected human fibroblasts with scrambled siRNA as a negative control (NC) or siRNAs selectively targeting PPARα for 48 h. Cells were subjected to serum-starvation for 24 h. In PPARα siRNA-transfected fibroblasts, PPARα mRNA level was significantly reduced compared to those of NC siRNA-transfected fibroblasts (to 48.8±0.7%; *p*<0.001; n = 3) (**Fig 4A**). Moreover, catalase mRNA expression in PPARα siRNA-transfected fibroblasts was also significantly reduced by 29.5% (*p*<0.05; n = 3) (**Fig 4A**). These results demonstrate that PPARα knockdown can lead to the reduction of catalase expression. We subsequently examined the effect of PPARα knockdown on UV-induced procollagen decrease and MMP-1 expression. After siRNA transfection and serum starvation, human skin fibroblasts were treated with UV in the absence or presence of Wy14643 treatment. Procollagen mRNA level, decreased by UV, recovered with Wy14643 treatment (100 μM) in NC siRNA-transfected, but not in PPARα siRNA-transfected, fibroblasts. Furthermore, UV-induced upregulation of MMP-1 mRNA was decreased with Wy14643 treatment in NC siRNA-transfected, but not in PPARα siRNA-transfected fibroblasts (**Fig 4B**). By western blot analysis, we confirmed the lack of Wy14643 effects on procollagen and MMP-1 expressions modulated by UV in fibroblasts transfected with PPARα siRNA (**Fig 4C**). Taken together, these results indicated that the effects of Wy14643 on UV-induced suppression of procollagen and upregulation of MMP-1 expression were mediated mainly by ligand-activated PPARα in human dermal fibroblasts.

### PPARα agonist increased the expression of procollagen and catalase while decreased the expression of MMP-1 and ROS in aged human dermal fibroblasts

**Fig 5:**
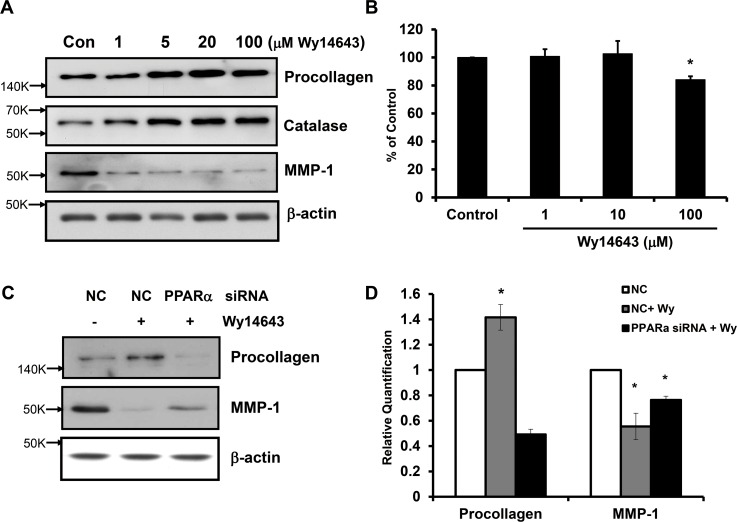
PPARα activator increased the expression of procollagen and catalase protein while decreased the expression of MMP-1 and ROS in aged human dermal fibroblasts. **(a)** Wy14643 (1–100 μM) was treated for 72 h to human dermal fibroblasts obtained from forearm skin of elderly subjects (mean age 78.6 year; n = 3). Procollagen and MMP-1 levels were measured using the culture media, and catalase and β-actin expression was using cell lysates by western blotting. The western bands are representative of three separate experiments. **(b)** Aged fibroblasts were treated with Wy14643 for 72 h, and cells were treated with 2,7-dichlororofluorescein diacetate (DCFDA) for 30 min, and assayed using a fluorescence reader. Data are presented as means±SEM (n = 6), *p<0.05 vs. control. **(c)** Aged fibroblasts were transfected with scrambled siRNA as a negative control (NC), or PPARα siRNA for 48 h. The transfected cells were serum starved for 24 h, and then, treated with 1–100 μM Wy14643 for 72 h. The western bands are representative of three separate experiments. **(d)** Procollagen and MMP-1 mRNA expression was measured by qPCR. Data are presented as mean±SEM (n = 3), *p<0.05, vs. cells transfected with NC siRNA.

As shown in **Fig 1A**, we demonstrated the reduction of PPARα mRNA expression in forearm skin of elderly individuals. Thus, we treated fibroblasts obtained from forearm skin of elderly individuals with Wy14643 in order to confirm the effect of a PPARα agonist in aged fibroblasts. As expected, procollagen and catalase protein levels were increased in a dose-dependent manner with Wy14643 treatment. In contrast, MMP-1 expression was decreased in a dose-dependent manner with Wy14643 in aged fibroblasts (**Fig 5A**). Wy14643 (100 μM) was also able to significantly reduce intracellular ROS level (*p*<0.05; n = 6) (**Fig 5B**). These results suggest that the induction of catalase by ligand-activated PPARα led to ROS reduction, which may contribute to the increased procollagen and decreased MMP-1 expressions in aged fibroblasts. In PPARα siRNA-transfected aged fibroblasts, Wy14643-mediated increase in procollagen level and decrease in MMP-1 expression were abrogated (**Fig 5C and 5D**). These results suggest that the effect of Wy14643 was also mediated by ligand-activated PPARα in aged fibroblasts, consistent with results from UV-irradiated fibroblasts.

### Topical application of Wy14643 prevented the induction of MMP-13, COX-2, IL-1β, and IL-6 expressions and the reduction of catalase activity by UV in hairless mouse skin

**Fig 6:**
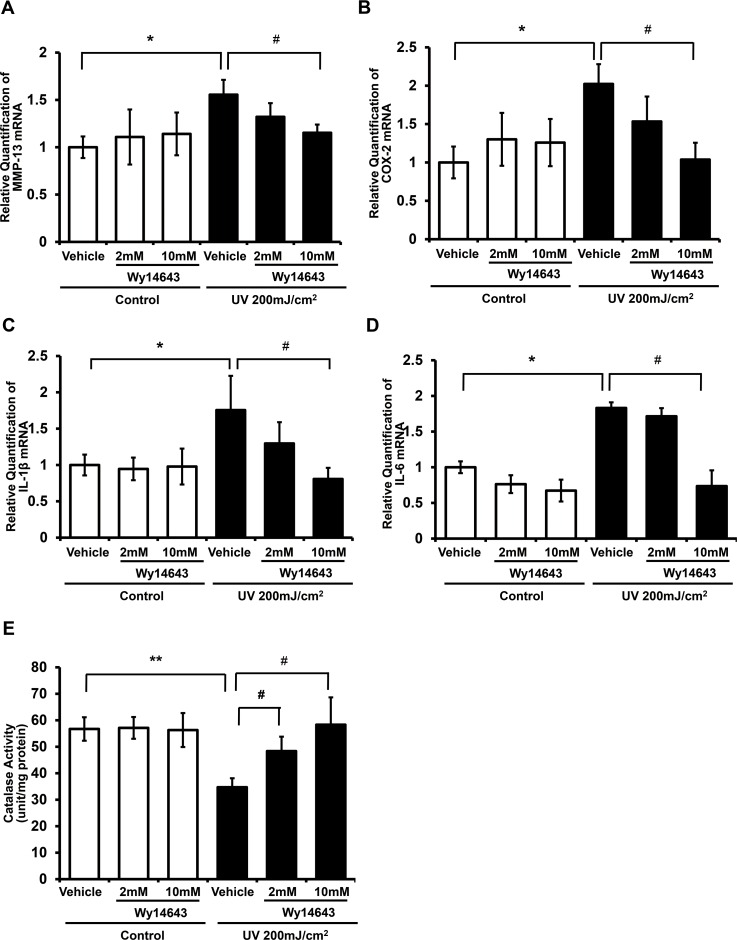
Topical application of Wy14643 prevented the induction of MMP-13, COX-2, IL-1β, and IL-6 expressions and the reduction of catalase activity by UV in hairless mouse skin *in vivo*. Dorsal skin of HR-1 hairless mice was topically treated with vehicle (ethanol:polyethylene glycol = 30:70) only or Wy14643 (2 mM or 10 mM), at 24 h prior to, immediately, and 24 h following UV irradiation. Skin biopsy was carried out at 48 h after UV irradiation. **(a)** MMP-13, **(b)** COX-2, **(c)** IL-1β and **(d)** IL-6 mRNA expression was quantified by qPCR. **(e)** Catalase activity was measured using total soluble protein by a spectrophotometric method. Catalase enzyme unit is equivalent to 1 μmol of substrate disappearance or product formation per min. Data are presented as mean±SEM (n = 8 for each group). *p<0.05, **p<0.01 vs. vehicle only; #*p*<0.05 vs. vehicle-treated UV-irradiated skin.

To investigate whether a PPARα ligand can prevent UV irradiation-induced damage in mouse skin *in vivo*, we assessed effects of Wy14643 on MMP-13, COX-2, IL-1β, and IL-6 mRNA expressions induced by UV using qPCR. We found that UV irradiation significantly enhanced the expression of MMP-13, COX-2, IL-1β, and IL-6 mRNA levels (155.5±15.7%, 202.3±25.7%, 175.5±47.1%, and 183.1±7.9%, respectively relative to the non-UV irradiated control) (**Fig 6A–6D**). However, topical application of 10 mM Wy14643 significantly attenuated the upregulation of MMP-13, COX-2, IL-1β, and IL-6 by UV (*p*<0.05; n = 8). In order to evaluate the effect of Wy14643 on catalase expression, we measured catalase activity in hairless mouse skin. In hairless mice, acute UV irradiation significantly decreased the catalase activity by 38.8% compared to the non-UV irradiated control group (*p*<0.01; n = 8). Catalase activity recovered to the control level in the 10 mM Wy14643-treated group (168% relative to that of the vehicle-treated UV-exposed group; *p*<0.05; n = 8) (**Fig 6E**). Taken together, these results demonstrate the protective effect of ligand-activated PPARα against UV-induced skin responses in hairless mice.

## Discussion

PPARα has been shown to play important roles in age-related inflammatory diseases and skin aging [30, 31]. It is known that liver and heart of aged animals exhibit a reduction in the expression of PPARα and its regulated genes [32, 33]. PPARα-null mice were more sensitive to kidney damage following ischemia-reperfusion and had reduced life span [34, 35]. However, little is known about the expression and function of PPARα related to aging in human tissues. In this study, we demonstrated the downregulation of PPARα mRNA expression in aged and photoaged human skin *in vivo*. Moreover, acute UV irradiation decreased PPARα expression in human skin *in vivo*. Catalase, a known PPARα target gene, is a potent antioxidant against ROS and oxidative stress [24]. Previously, we found that catalase in human skin was decreased with aging and photoaging, suggesting that the induction of catalase expression can be an effective approach for the prevention and treatment of aging and photoaging of the human skin [15, 16].

Here, we hypothesized that age-dependent reduction of PPARα in the skin may result in elevated oxidative stress due to reduced catalase expression, leading to skin aging. Therefore, we investigated whether a PPARα activator can increase catalase expression in the skin, and consequently attenuate skin aging and UV-induced skin responses. Although the activation of PPARβ/δ or PPARγ has been associated with an altered procollagen expression [36–39], the effect of PPARα agonist on procollagen expression in the skin is not fully understood. In the current study, we demonstrated that the activation of PPARα by its ligand can increase the level of catalase mRNA, protein, and enzyme activity, inhibit ROS generation, and prevent MMP-1 induction and collagen destruction in UV-irradiated human dermal fibroblasts. It was reported that PPARα activation can stimulate catalase expression in human hepatocytes [40] and spleens of aged mice [41]. Consistent with findings in UV-irradiated cells, Wy14643 treatment of aged dermal fibroblasts was found to increase catalase expression and reduce ROS production, which led to increased procollagen expression and decreased MMP-1 expression. Conversely, fibroblasts transfected with PPARα siRNA showed reduced catalase expression and an abrogation of Wy14643’s protective effect in modulating UV-induced MMP-1 and procollagen levels. These results indicated that effects of Wy14643 in the induction of catalase and collagen maintenance in the skin were mediated by PPARα. Earlier studies showed the ability of PPARα agonists to reverse the transforming growth factor-α-induced MMP-9 expression in human keratinocytes [42]. More recently, novel PPARα activators and a PPARα/γ dual agonist from natural or synthetic origin have been reported to exert a protective effect against UV- and TNF-α-induced inflammation and matrix damage [25, 43, 44].

Importantly, we demonstrated that a topical application of Wy14643 was able to ameliorate UV-induced reduction of catalase activity in hairless mouse skin *in vivo*. In addition, Wy14643 was able to limit the production of MMP-13, COX-2, IL-1β, and IL-6 induced by UV in mouse skin. PPARα deficient-mice have been reported to exhibit exacerbated inflammation in a mouse model of atopic dermatitis, and the expression of PPARα is reduced in the lesional skin of atopic dermatitis [45]. Topical treatment regimen consisting of PPARα activator showed potent anti-inflammatory effects in murine models of atopic dermatitis [45, 46] and in irritant and allergic contact dermatitis, which were associated with reduction in pro-inflammatory cytokines TNF-α and IL-1α [47]. PPARα agonists were also shown to reverse the induction of inflammatory cytokines such as IL-6 and IL-8 mediated by UVB in human keratinocytes [48]. Consistent with these reports, we demonstrated the anti-inflammatory potentials of a PPARα agonist, Wy14643, in UV-irradiated mouse skin.

There have been numerous attempts to develop novel agents against skin aging. Treatments using antioxidants, MMPs inhibitors, collagen stimulators, as well as anti-inflammatory agents are feasible approaches to alleviate skin damage induced by UV in aged skin. Our results indicated that the upregulation of catalase using a PPARα agonist is a promising therapeutic strategy against aging and inflammation of the human skin. The mechanism behind PPARα downregulation in aged or UV-irradiated skin remains to be elucidated. Further studies should focus on the underlying mechanisms of PPARα downregulation by photoaging, intrinsic aging, and acute UV irradiation.

## Supporting Information

S1 Fig Original uncropped and unadjusted blots in figures. (PPTX)
