# Supplementary material for: Activation of Peroxisome Proliferator-Activated Receptor Alpha Improves Aged and UV-Irradiated Skin by Catalase Induction
Source: PLoS One. 2016 Sep 9;11(9):e0162628. doi: 10.1371/journal.pone.0162628 (PMC5017777; doi:10.1371/journal.pone.0162628)
Supplement: S1 Fig — (PPTX) [file pone.0162628.s001.pptx]

## Slide 1
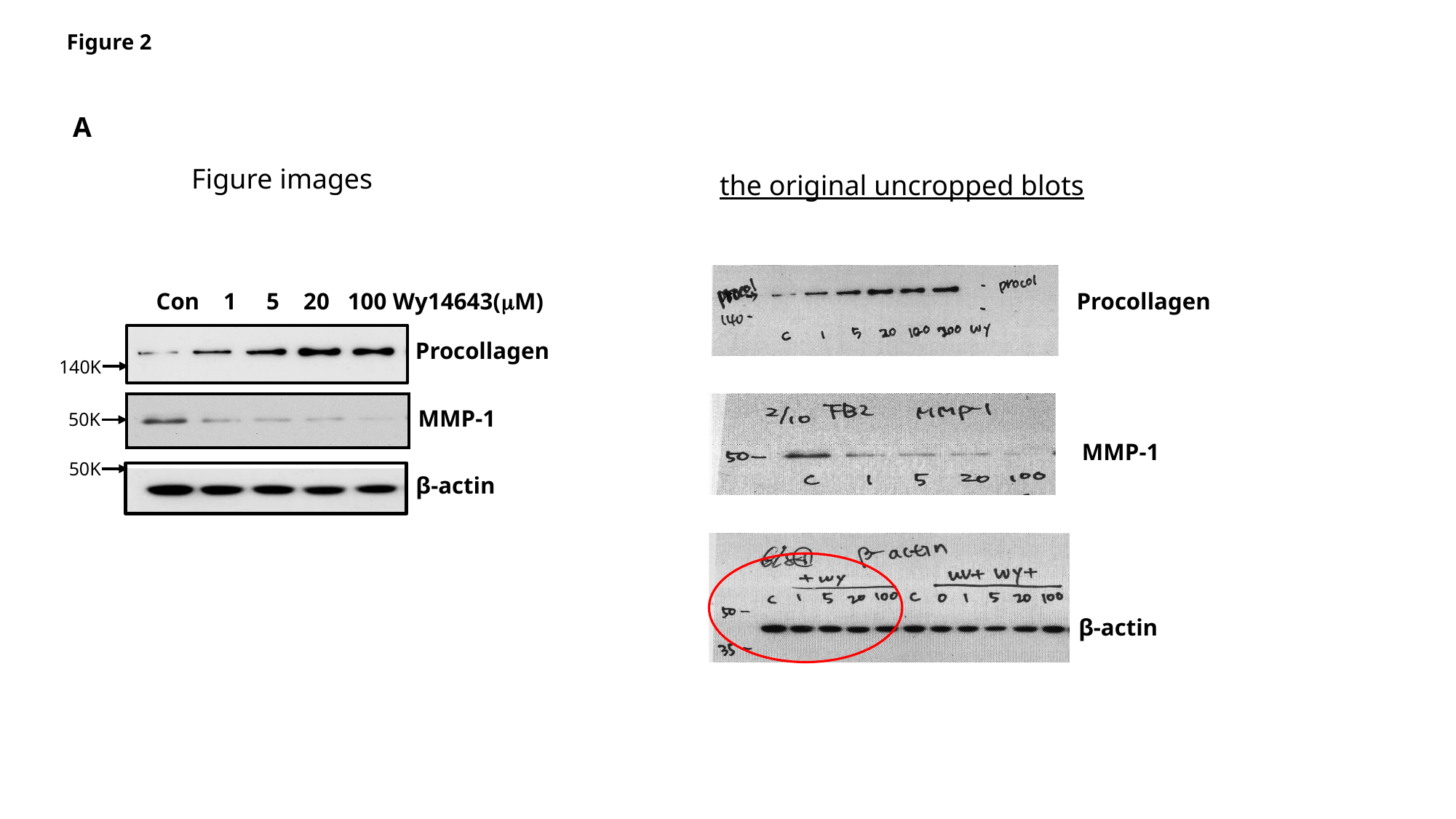

Figure 2
A
Figure images
the original uncropped blots
Con 1 5 20 100 Wy14643(μM)
Procollagen
Procollagen
140K
MMP-1
 50K
MMP-1
 50K
β-actin
β-actin

## Slide 2
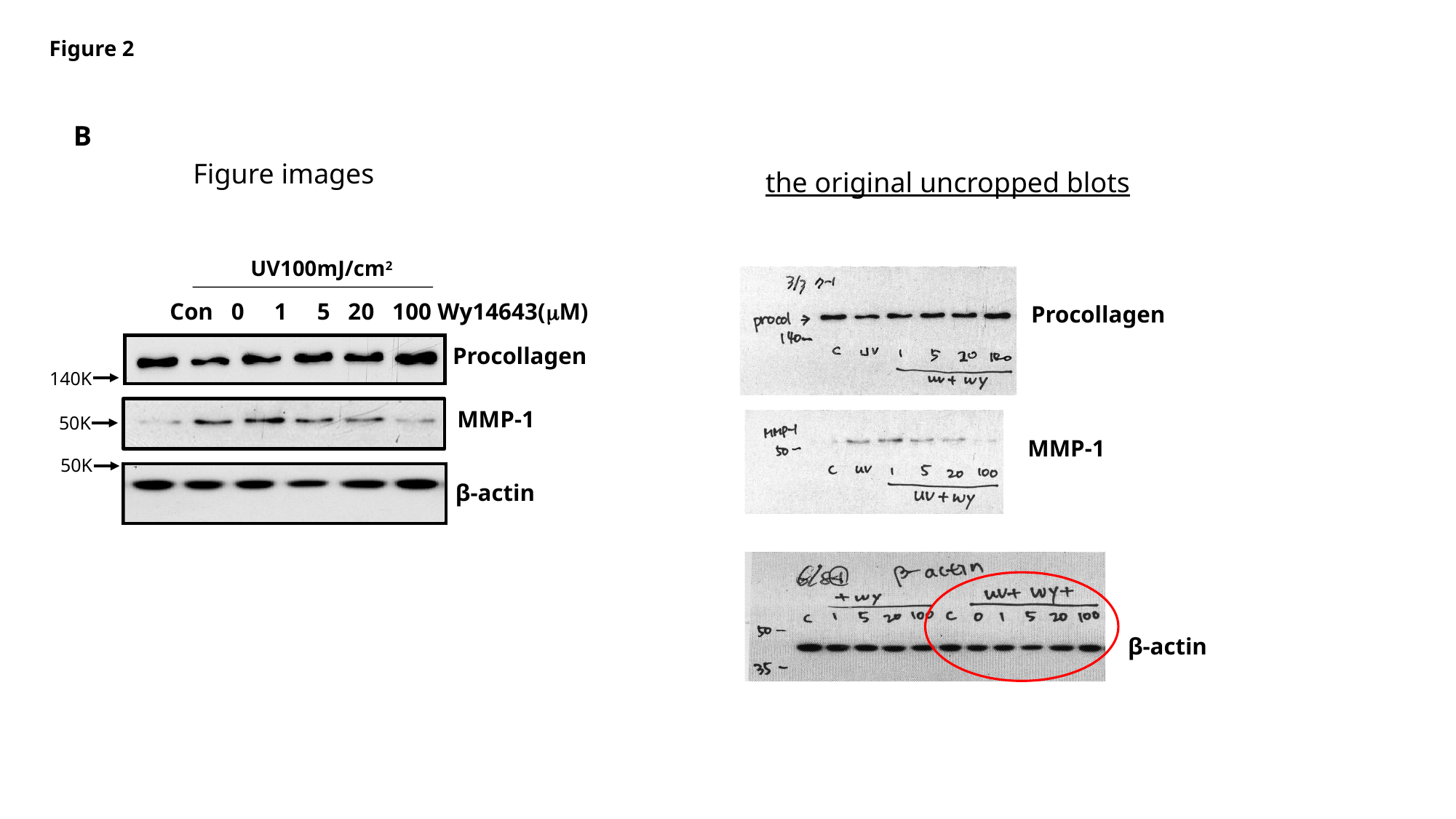

Figure 2
B
Figure images
the original uncropped blots
UV100mJ/cm2
Con 0 1 5 20 100 Wy14643(μM)
Procollagen
Procollagen
140K
MMP-1
 50K
MMP-1
 50K
β-actin
β-actin

## Slide 3
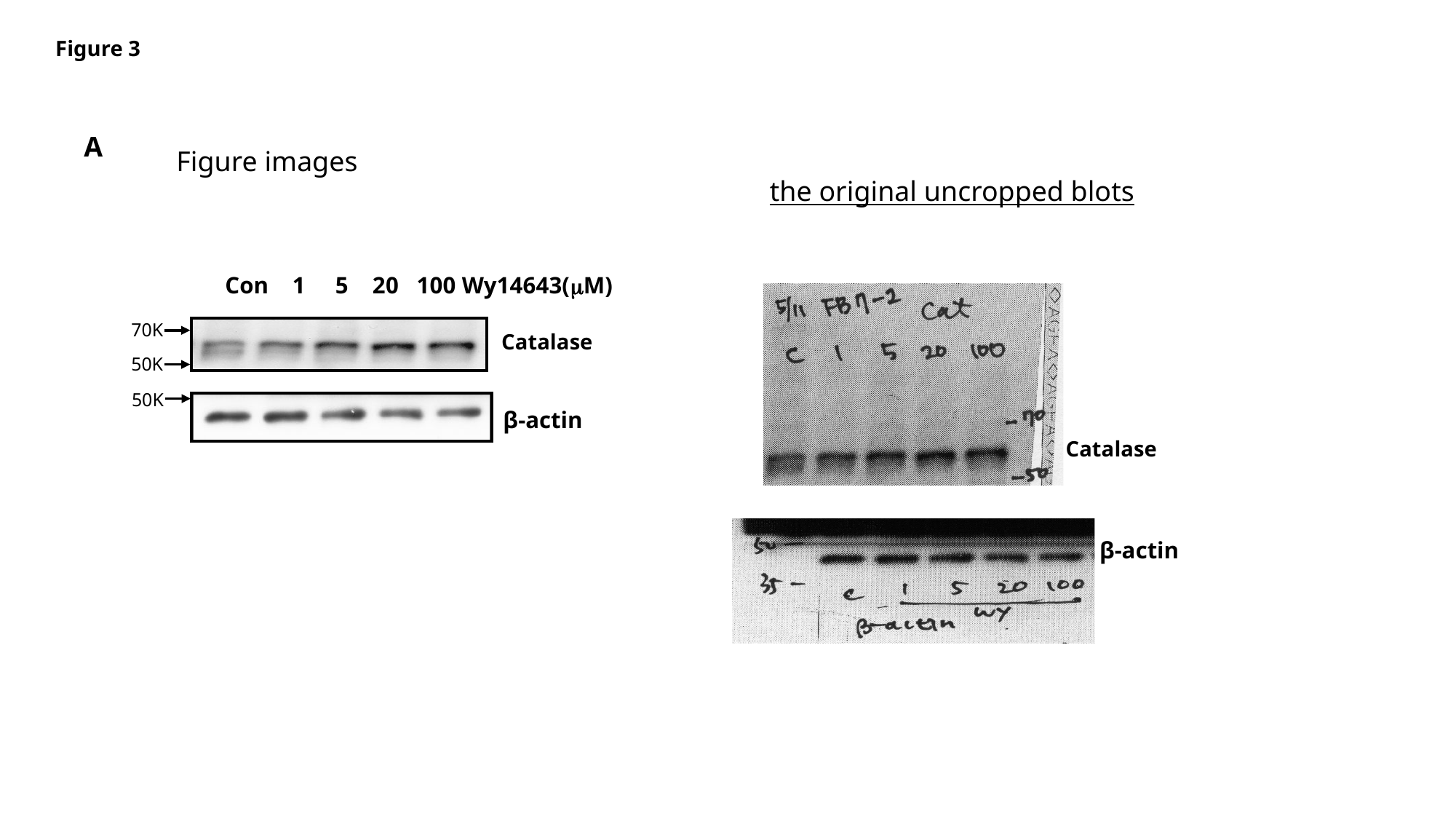

Figure 3
A
Figure images
the original uncropped blots
Con 1 5 20 100 Wy14643(μM)
 70K
Catalase
 50K
 50K
β-actin
Catalase
β-actin

## Slide 4
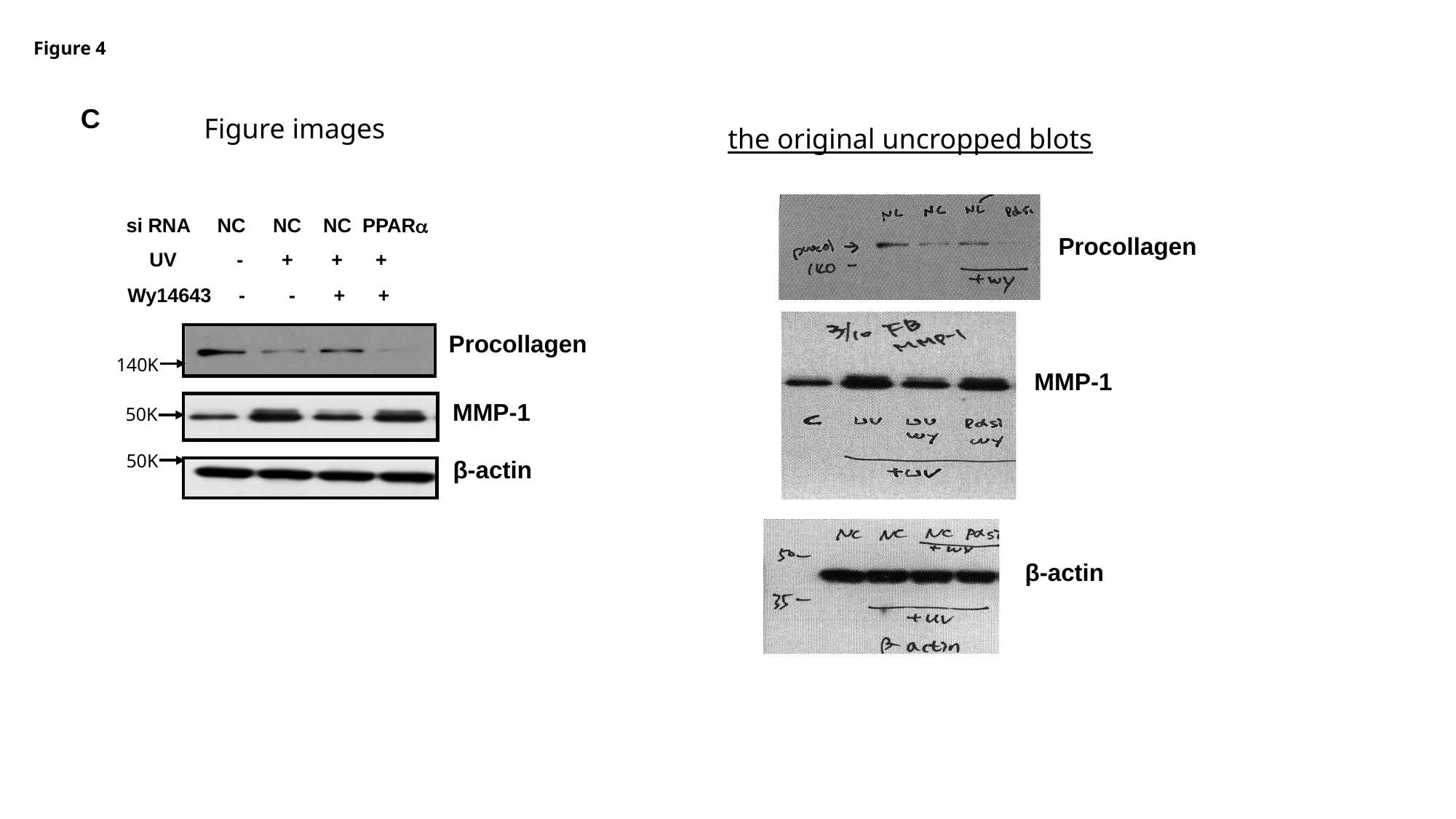

Figure 4
C
Figure images
the original uncropped blots
si RNA NC NC NC PPARα
Procollagen
UV - + + +
Wy14643 - - + +
Procollagen
140K
MMP-1
MMP-1
 50K
 50K
β-actin
β-actin

## Slide 5
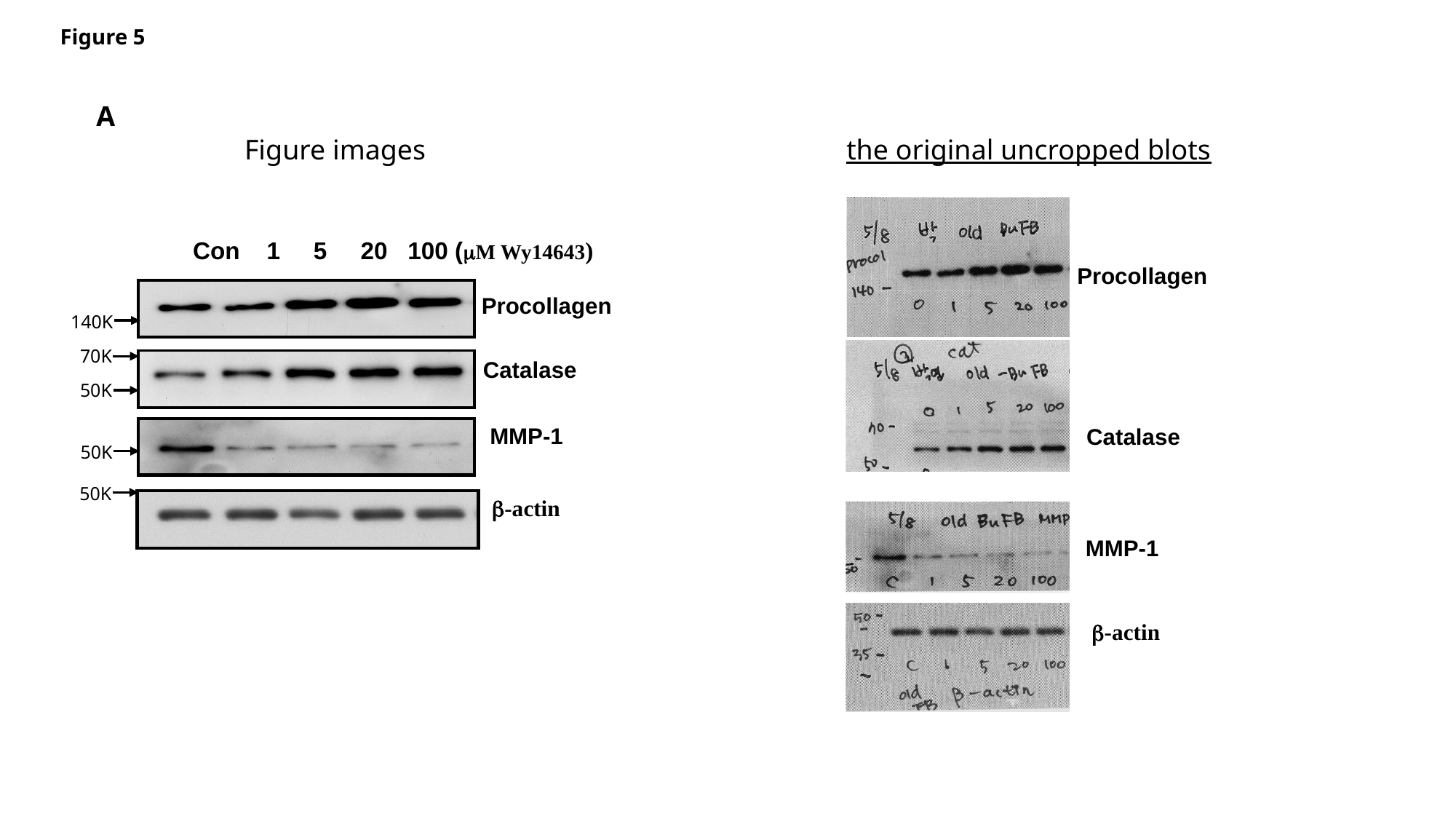

Figure 5
A
Figure images
the original uncropped blots
Con 1 5 20 100 (μM Wy14643)
Procollagen
Procollagen
140K
 70K
Catalase
 50K
MMP-1
Catalase
 50K
 50K
β-actin
MMP-1
β-actin

## Slide 6
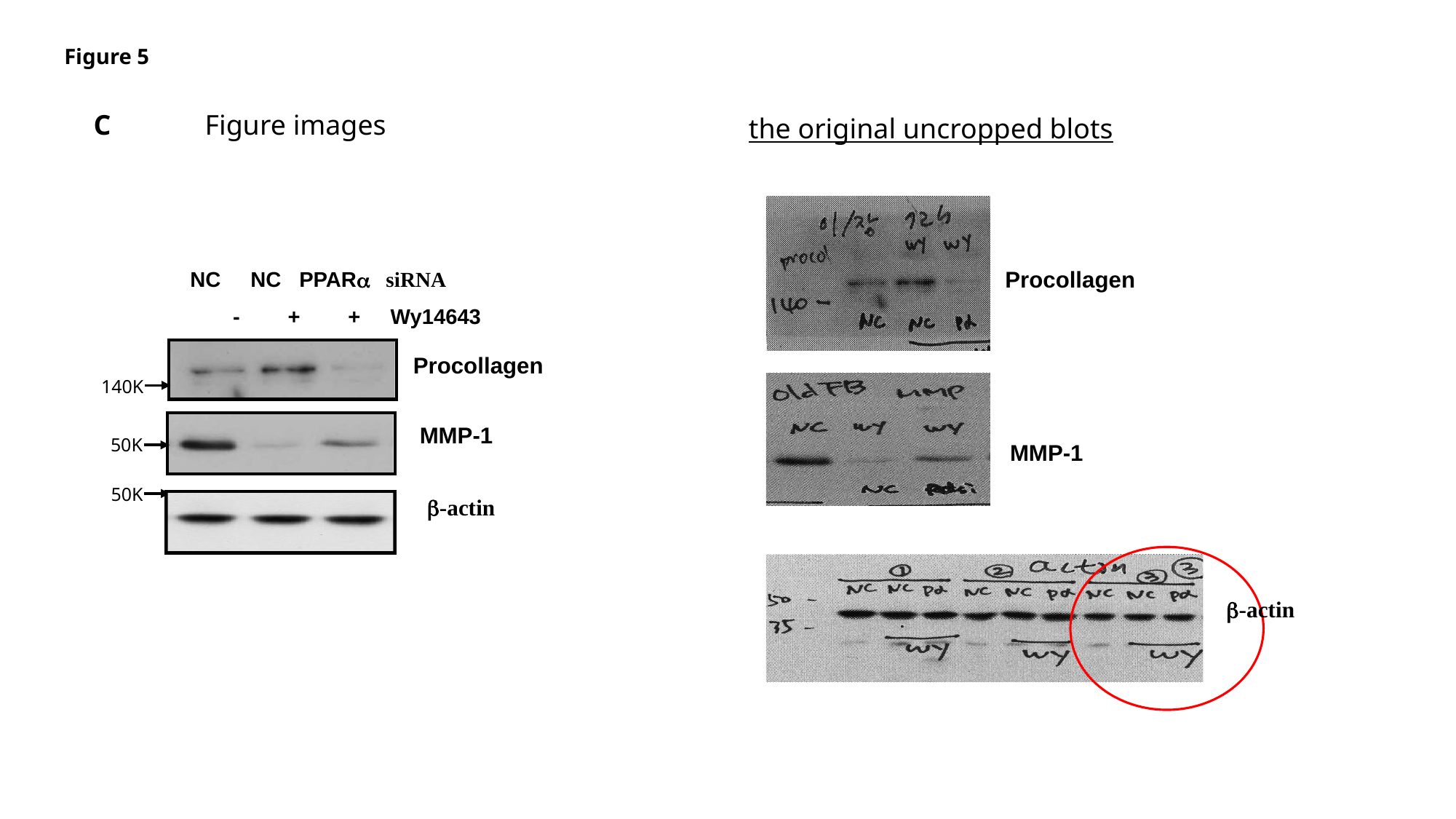

Figure 5
C
Figure images
the original uncropped blots
Procollagen
 NC NC PPARα siRNA
- + + Wy14643
Procollagen
140K
MMP-1
 50K
MMP-1
 50K
β-actin
β-actin
